# Complex Formation with Monomeric α-Tubulin and Importin 13 Fosters c-Jun Protein Stability and Is Required for c-Jun’s Nuclear Translocation and Activity

**DOI:** 10.3390/cancers11111806

**Published:** 2019-11-17

**Authors:** Melanie Kappelmann-Fenzl, Silke Kuphal, Rosemarie Krupar, Dirk Schadendorf, Viktor Umansky, Lily Vardimon, Claus Hellerbrand, Anja-Katrin Bosserhoff

**Affiliations:** 1 Institute of Biochemistry (Emil-Fischer Center), Friedrich-Alexander University, Erlangen-Nürnberg, 91054 Erlangen, Germanysilke.kuphal@fau.de (S.K.); claus.hellerbrand@fau.de (C.H.); 2 Faculty of Applied Health Care Sciences, University of Applied Science Deggendorf, 94469 Deggendorf, Germany; 3 Pathology of the University Medical Center Schleswig-Holstein, Campus Lübeck and Research Center Borstel, Leibniz Center for Medicine and Biosciences, 23566 Lübeck, Germany; rkrupar@fz-borstel.de; 4 Department of Dermatology, University Duisburg-Essen, 45355 Essen, Germany; dirk.schadendorf@uk-essen.de; 5 Department of Dermatology, Venereology and Allergology, University Medical Center Mannheim, Ruprecht-Karl University of Heidelberg, 69117 Heidelberg, Germany; v.umansky@dkfz.de; 6 Department of Biochemistry and Molecular Biology, Tel Aviv University, 69978 Tel Aviv, Israel; vardi@post.tau.ac.il; 7 Comprehensive Cancer Center (CCC) Erlangen-EMN, 91054 Erlangen, Germany

**Keywords:** AP1, c-Jun, transcription factor, tubulin, importin

## Abstract

Microtubules are highly dynamic structures, which consist of α- and β-tubulin heterodimers. They are essential for a number of cellular processes, including intracellular trafficking and mitosis. Tubulin-binding chemotherapeutics are used to treat different types of tumors, including malignant melanoma. The transcription factor c-Jun is a central driver of melanoma development and progression. Here, we identify the microtubule network as a main regulator of c-Jun activity. Monomeric α-tubulin fosters c-Jun protein stability by protein–protein interaction. In addition, this complex formation is necessary for c-Jun’s nuclear localization sequence binding to importin 13, and consequent nuclear import and activity of c-Jun. A reduction in monomeric α-tubulin levels by treatment with the chemotherapeutic paclitaxel resulted in a decline in the nuclear accumulation of c-Jun in melanoma cells in an experimental murine model and in patients’ tissues. These findings add important knowledge to the mechanism of the action of microtubule-targeting drugs and indicate the newly discovered regulation of c-Jun by the microtubule cytoskeleton as a novel therapeutic target for melanoma and potentially also other types of cancer.

## 1. Introduction

Melanoma is a highly aggressive type of skin cancer that originates from pigment producing melanocytes [1]. In recent decades, many altered pathways regulating the development and progression of melanoma and the high migratory and invasive potential of melanoma cells have been identified, but a detailed molecular understanding of this disease is largely lacking [2,3]. Despite the recent spectacular improvements in targeted melanoma treatment (i.e., BRAF (B-Raf Proto-Oncogene, Serine/Threonine Kinase) and MEK (mitogen-activated protein kinase kinase enzymes) inhibitors or immunotherapies), more than half of the patients will be in treatment failure and chemotherapy may still be important in the palliative treatment of refractory, progressive, and relapsed melanoma [4,5]. 

Microtubule-targeting agents such as paclitaxel have been used in chemotherapy against metastatic melanoma for decades [6,7]. They interfere with intracellular transport, inhibit eukaryotic cell proliferation, and promote cell death by suppressing microtubule dynamics [8].

C-Jun is a member of the activator protein 1 (AP-1) transcription factor family and its activity is known to play an important role in melanoma development and progression [9,10,11]. Recent studies further indicated a crucial role of the c-Jun/AP-1 transcription factor complex in therapy resistance, including checkpoint inhibition [12,13] and immune response [14]. Therefore, the identification of the molecular mechanisms leading to c-Jun protein expression and thus AP-1 activation in melanoma is of very high clinical interest.

In this study, we show for the first time that microtubule dynamics significantly influence AP-1 activity by regulating the c-JUN protein. Furthermore, we detect an interaction between monomeric α-tubulin and c-JUN protein, which stabilizes the transcription factor, influences its transport to the nucleus, and subsequently affects c-JUN and thus AP-1 activity in malignant melanoma. Moreover, we newly demonstrate that microtubule-targeting agents effectively inhibit c-Jun/AP-1 transcription factor activity in melanoma.

## 2. Results and Discussion

Microtubules are dynamic filamentous cytoskeletal proteins composed of tubulin, and until the advent of targeted therapy, microtubules were the only alternative to DNA as a therapeutic target in cancer [6,15]. Interestingly, paclitaxel (PX), which promotes assembly and causes increased microtubule density and bundling [16], and nocodazole (NX), which promotes disruption of microtubule assembly [17], led to opposed changes in the transcriptional activity of AP-1 in melanoma cells. Microtubule dynamics are known to play a crucial role during tumor progression and development, and some recent studies have focused on the function of microtubule alterations in cancer cells [18], but the detailed molecular mechanisms have not been investigated so far. 

**Figure 1:**
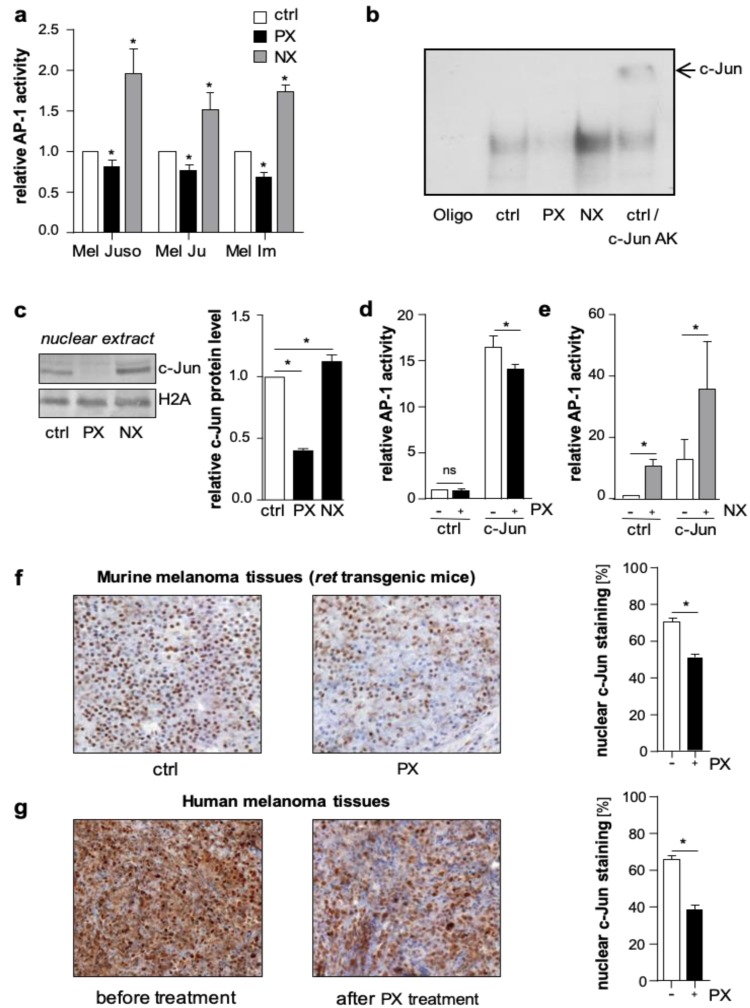
Microtubule-targeting drugs (paclitaxel and nocodazole) affect activator protein 1 (AP-1) activity in melanoma cells in vitro and in vivo. (**a**) Analyses of AP-1 luciferase reporter gene activity of human melanoma cells Mel Juso, Mel Ju, and Mel Im after treatment with the microtubule-stabilizing agent paclitaxel (PX; 5 µM) or nocodazole (NX; 30 µM), an agent promoting disruption of microtubule assembly. Control cells (ctrl) were treated with solvent DMSO (Dimethyl sulfoxide). (**b**) Electrophoretic mobility shift assays (EMSA) with nuclear extracts of PX or NX treated Mel Ju cells using the classical AP-1 consensus sequence (Oligo). Supershift experiments with an anti-c-Jun antibody demonstrate the direct involvement of c-Jun in the AP-1–DNA-binding complex. (**c**) Western blot analyses and densitometry of c-Jun in nuclear extracts of PX and NX treated Mel Juso cells and control cells (ctrl). Histone H2A was used as the loading control. (**d**,**e**) AP-1 luciferase reporter gene activity in (**d**) PX and (**e**) NX treated Hmb2-5 cells transfected with a c-Jun expression plasmid (c-Jun) or empty vector (pCDNA3; ctrl). (**f**) Immunohistochemical analyses of c-jun in melanoma tissues from *ret* transgenic mice (*n* = 3) after the treatment with PX (15 mg/kg) or PBS (ctrl). PX application was performed at day 0 and 5, and mice were sacrificed on day 7. (**g**) Immunohistochemical analyses of c- Jun in melanoma tissues from five patients before and after PX treatment. (**f**,**g**) The right panels depict the quantification (mean ± s.e.m.) of c-Jun positive nuclei per viewing field. (*: *p <* 0.05; ns: not significant).

In luciferase reporter gene assays, treatment with NX increased, whereas PX decreased AP-1 activity in primary (Mel Juso) and metastatic (Mel Ju, Mel Im) human melanoma cells (Figure 1a and Figure S1a). In line with this, electrophoretic mobility shift assays (EMSA) revealed a PX-dependent decrease and an NX-dependent increase in the direct DNA-binding capacity of AP-1 to the 12-O-tetradecanoylphorbol-13-acetate (TPA) responsive element 5′-TGAG/CTCA-3′, the classical AP-1 consensus sequence (Figure 1b and Figure S1b). Supershift experiments with an anti-c-Jun antibody confirmed the direct involvement of c-Jun in the AP-1-DNA binding complex in melanoma cells (Figure 1b and Figure S1b). To analyze the effects of microtubule-targeting drugs on nuclear c-Jun protein levels in melanoma cells, we performed western blot analyses. PX treatment resulted in a decreased accumulation of c-Jun protein in the nucleus, whereas NX treatment led to a nuclear enrichment of c-Jun (Figure 1c).

To further study the regulation of c-Jun by microtubule dynamics, we applied Hmb2-5 cell clones, a model system resembling melanocytes and almost lacking c-Jun expression [19,20]. In accordance with the lack of c-Jun, luciferase reporter gene analyses showed low basal AP-1 activity in Hmb2-5 cell clones, and PX treatment did not result in further reduced activity (Figure 1d). However, NX treatment significantly induced basal AP-1 activity in these cells (Figure 1e). Furthermore, transfection with a c-Jun expression construct led to a strong induction of AP-1 activity, which significantly decreased after PX (Figure 1d**)** and increased after NX treatment (Figure 1e). These results suggest that microtubules regulate the activity of AP-1 in melanoma cells in a c-Jun-specific manner. In line with our results, Ishiguro and colleagues showed that α-tubulin (TUB1A) functions as an adaptor for the nuclear transport of the transcription factor NFAT (Nuclear factor of activated T-cells) by importin β to modulate immune responses [21]. Moreover, the tumor suppressor CYLD (cylindromatosis) was reported to be associated with microtubules. Furthermore, it was demonstrated that CYLD enhances tubulin polymerization into microtubules by lowering the critical concentration for microtubule assembly [22]. Additionally, the transcription factor HIF-1α was also regulated by microtubule dynamics. Here, the polymerized microtubules were critically involved in the nuclear trafficking and transcriptional activity of HIF-1α [23]. In this study, we described a novel regulatory mechanism for c-Jun stabilization by the c-Jun/α-tubulin interaction. 

To further verify whether microtubule density influences the nuclear accumulation of c-Jun in vivo, we treated *ret* transgenic melanoma bearing mice [24] twice (day 0 and day 5) with PX (15 mg/kg body weight) or vehicle (Phosphate buffered saline (PBS) control group). Immunohistochemical analyses of murine melanoma tissues revealed less nuclear c-Jun accumulation in the PX group compared to control (Figure 1f). Also, in human melanoma tissues derived from five patients before and after PX treatment, immunohistochemistry confirmed that the nuclear c-Jun accumulation significantly declined after PX therapy (Figure 1g).

**Figure 2:**
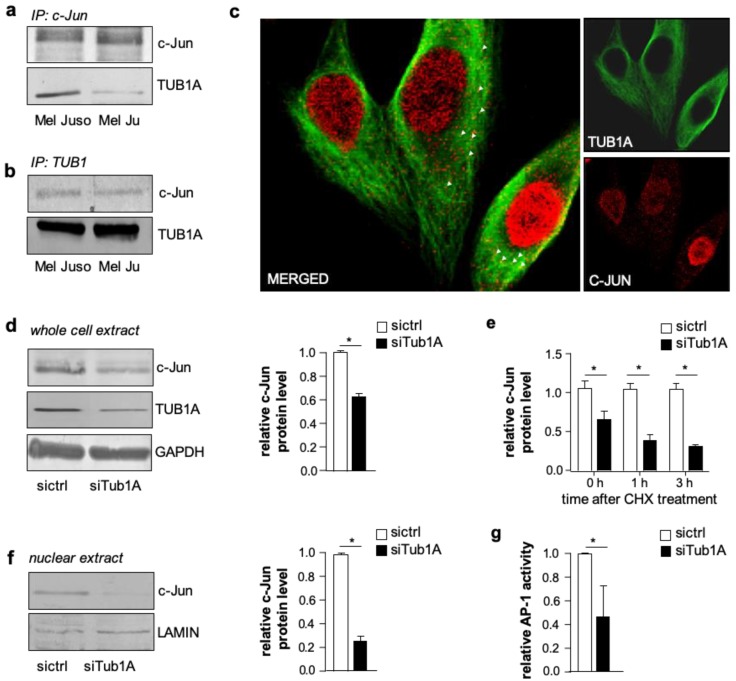
c-Jun protein interacts with TUB1A (Tubulin alpha chain) in melanoma cells and TUB1A affects AP-1 activity and stabilizes c-Jun protein. (**a**,**b**) Immunoprecipitation (IP) analyses of melanoma cell (Mel Juso, Mel Ju) lysates revealed co-precipitation of TUB1A with an (**a**) anti-c-Jun antibody and vice versa, (**b**) c-Jun with anti-TUB1A antibody. (**c**) Immunofluorescence analyses showed co-localization (white arrows) of c-Jun (red) and TUB1A (green) in the cytoplasm of melanoma cells. (**d**) Western blot analyses and densitometry of c-Jun and TUB1A in whole cell lysates of Mel Juso cells after TUB1A si-RNA (siTub1A) or control si-RNA (sictrl) transfection. GAPDH (Glyceraldehyde-3-Phosphate Dehydrogenase) was used as a loading control. The bar graph depicts the quantification of protein amounts (mean ± s.d.) of three independent experiments. (**e**) Analyses of c-Jun protein expression in TUB1A-suppressed (siTub1A) and control (sictrl) Mel Juso cells after cycloheximide (CHX) treatment showed a faster decline of c-Jun levels in siTub1A compared to control cells. The bar graph (mean ± s.d. of three western blot analyses) depicts c-Jun levels normalized to GAPDH. (**f**) Western blot analyses and densitometry of nuclear extracts of Mel Juso cells showed lower c-Jun protein levels in TUB1A-suppressed (siTub1A) compared to control (sictrl) cells. The bar graph depicts c-Jun levels of three western blot analyses relative to LAMIN (Lamin A/C), which was used as a loading control. (**g**) AP-1 luciferase reporter gene analyses showed reduced AP-1 activity in TUB1A-suppressed (siTub1A) Mel Juso cells compared to control (sictrl) cells. Bars show the means ± s.d. of three independent experiments; measurements were performed in replicates. (*: *p <* 0.05).

To further investigate the mechanism of c-Jun regulation via the cytoskeleton, we first examined whether there was a direct molecular interaction. However, co-sedimentation by ultra-centrifugal spin-down assays showed that there was no binding between c-Jun and polymerized microtubules (Figure S2). We next determined whether c-Jun interacted with monomeric TUB1A. The immunoprecipitation of c-Jun from whole melanoma cell lysates (Mel Juso and Mel Ju) and subsequent western blot analyses of TUB1A showed an interaction between c-Jun and TUB1A (Figure 2a; protein input depicted in Figure S3a). Conversely, immunoprecipitation with an anti-TUB1A antibody corroborated the association between c-Jun and monomeric TUB1A (Figure 2b; protein input depicted in Figure S3b). Confocal microscopy and immunofluorescence analyses confirmed the co-localization between c-Jun and TUB1A in the cytoplasm of melanoma cells (Figure 2c and Figure S3c). 

To further investigate the association between TUB1A and c-Jun, we analyzed the effect of TUB1A knockdown with si-RNA on c-Jun protein levels in melanoma cells. Western blot analyses demonstrated decreased c-Jun protein in TUB1A suppressed cells (siTub1A) compared to control (siCtrl) transfected cells (Figure 2d and Figure S4). After treatment with cycloheximide, an inhibitor of protein synthesis, c-Jun protein levels declined faster in TUB1A suppressed cells (Figure 2e), suggesting that TUB1A contributes to the stability of the c-Jun protein. Moreover, western blot analyses revealed decreased c-Jun protein levels in nuclear extracts of TUB1A suppressed compared to control cells (Figure 2f). Accordingly, luciferase reporter gene assays showed a significant decrease in AP-1 activity after silencing TUB1A compared to control-transfected cells (Figure 2g and Figure S5). These results suggest that TUB1A promotes the transcriptional activity of c-Jun. However, in an EMSA applying a classical AP-1 DNA-binding sequence and nuclear extracts of melanoma cells, supershift analyses demonstrated that there was no direct association of TUB1A with c-Jun at DNA-binding (Figure S6). In line with this, immunofluorescence staining of TUB1A and c-Jun showed no (co)localization of TUB1A to the nucleus of melanoma cells (Figure 2c). 

**Figure 3:**
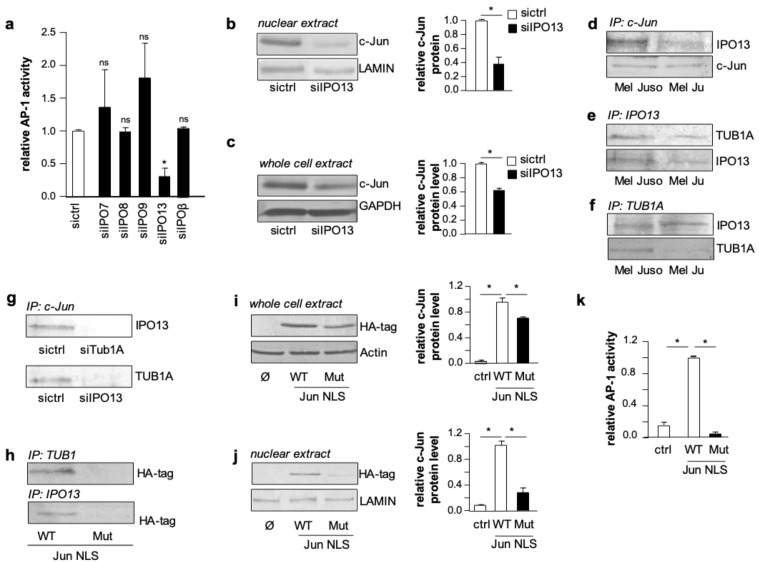
Stabilization of c-Jun by TUB1A influences nuclear import of c-Jun by IPO13. (**a**) AP-1 luciferase reporter gene activity in Mel Juso cells with si-RNA mediated suppression of several importins (siIPO7, siIPO8, siIPO9, siIPO13, siIPOβ) and cells transfected with control si-RNA (sictrl). The bar graph shows the means ± s.d. of three independent experiments. (*: *p <* 0.05 compared to sictrl; ns: not significant). (**b**,**c**) Analyses of c-Jun protein levels in (**b**) nuclear extracts and (**c**) whole cell extracts of melanoma cells with IPO13 suppression (siIPO13) and control cells (sictrl) by western blot and densitometry. GAPDH or LAMIN served as loading controls. (**d**–**f**) Immunoprecipitation (IP) analyses of whole cell lysates from Mel Juso and Mel Ju cells performing co-precipitation of (**d**) IPO13 using an anti-c-Jun antibody, (**e**) TUB1A using an anti-IPO13 antibody, and (**f**) IPO13 using an anti-TUB1A antibody. (**g**) Co-IP of Mel Juso cell lysates with si-RNA mediated suppression of TUB1A (siTub1A) or IPO13 (siIPO13) and sictrl using an anti-c-Jun-antibody. (**h**) Co-IP of extracts from Hmb2-5 cells transfected with wildtype JUN nuclear localization sequence (Jun NLS WT) or a Jun plasmid with mutated NLS (Jun NLS Mut) applying anti-TUB1A- or anti-IPO13-antibodies. Depicted are western blots using an anti-HA-tagged antibody for c-Jun detection. (**i**) Western blot analyses and densitometry showing the expression of HA-tagged c-Jun in Hmb2-5 protein lysates after transfection with Jun WT NLS or Jun MUT NLS. β-Actin served as a loading control. (**j**) Western blot analyses and densitometry showing the expression of HA-tagged c-Jun in Hmb2-5 nuclear extracts after transfection with Jun WT NLS or Jun MUT NLS. LAMIN served as a loading control. (**k**) AP-1 luciferase reporter gene activity of Hmb2-5 cells after transfection with Jun NLS WT or Jun NLS Mut. Bar graph shows the means ± s.d. of three independent experiments. (*: *p <* 0.05).

This prompted us to investigate whether TUB1A affects the nuclear import of c-Jun. Different importins (IPOs) have been shown to play a role in the nuclear import of c-Jun in other cancer entities [25,26]. Hence, we performed AP-1 luciferase reporter gene assays in melanoma cells with si-RNA suppression of five different importins (siIPO7, siIPO8, siIPO9, siIPO13, and siIPOβ). Interestingly, only IPO13 suppression had a significant effect on AP-1 activity (Figure 3a and Figure S7), indicating this importin as a so far unknown mediator of c-Jun activity in melanoma cells. Western blot analyses of nuclear extracts confirmed the impact of IPO13 on c-Jun regulation (Figure 3b). Notably, we also observed reduced c-Jun protein levels in total protein lysates of melanoma cells with IPO13 suppression compared to control cells (Figure 3c). Therefore, we speculated that IPO13 is involved in stabilizing c-Jun for nuclear import. To address this hypothesis, we performed co-immunoprecipitation analyses applying antibodies directed against IPO13, c-Jun, or TUB1A in total cellular lysates of melanoma cells. After controlling the input protein amounts (Figure S3), we determined an association between IPO13 and c-Jun in precipitates extracted with an anti-c-Jun antibody (Figure 3d). Furthermore, we detected an association between IPO13 and TUB1A in precipitates extracted with an anti-IPO13 antibody (Figure 3e). Finally, the application of an anti-TUB1A antibody showed that IPO13 and TUB1A can also be co-precipitated (Figure 3f). Control experiments confirmed the specificity of the IPO13, c-Jun, and TUB1A interactions (Figure S8). These results implicate that c-Jun protein utilizes TUB1A for its stabilization and binds to IPO13 for nuclear import. 

To identify the nuclear import association complex, we knocked down TUB1A or IPO13 in melanoma cells and carried out co-immunoprecipitation analyses with an anti-c-Jun antibody. These analyses revealed that in TUB1A- or IPO13-knockdown cells the interaction between c-Jun/IPO13 and c-Jun/TUB1A was no longer detectable (Figure 3g; protein input is depicted in Figure S3). This result indicates that the nuclear activity of c-Jun in melanoma cells depends on the presence of both TUB1A, which stabilizes and transports c-Jun, and IPO13, which is required for the nuclear import of c-Jun. 

Next, we analyzed whether the nuclear localization sequence (NLS) of c-Jun plays a role in the c-Jun/TUB1A or c-Jun/IPO13 interaction. To address this question, we transfected Hmb2-5 cell clones with hemagglutinin (HA)-tagged c-Jun expression plasmids carrying either the wild type NLS or a mutated form. Co-immunoprecipitation experiments with cell lysates using anti-TUB1A or anti-IPO13 antibodies showed that mutation of the c-Jun NLS binding resulted in a loss of both TUB1A and IPO13 interaction with c-Jun (Figure 3h). Western blot analyses revealed significantly lower c-Jun protein levels in both total cell lysates (Figure 3i) and nuclear extracts (Figure 3j) of cells transfected with the mutated c-Jun NLS. In line with this, AP-1 luciferase reporter gene assays revealed that the c-Jun expression construct with the mutated NLS is not able to induce AP-1 activity in Hmb2-5 cell clones (Figure 3k). These findings indicate that the NLS is required for the stabilization as well as the nuclear import and transcriptional activity of c-Jun.

**Figure 4:**
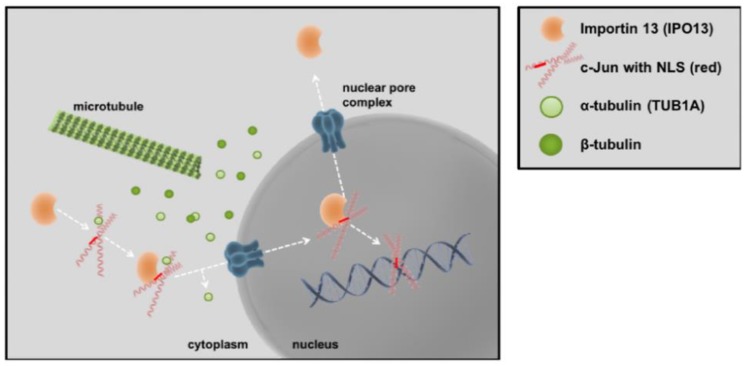
Schematic overview of the c-Jun/TUB1A/IPO13 complex in melanoma cells. Monomeric α-tubulin (TUB1A) stabilizes the transcription factor c-Jun for nuclear transport via importin 13 (IPO13). The complex assembly of c- Jun, TUB1A, and IPO13 occurs in a nuclear localization sequence (NLS)- dependent manner, however, TUB1A remains in the cytoplasm whereas c-Jun translocates into the nucleus via IPO13, and hence, affects AP-1 activity in melanoma cells.

Together, our results describe a novel regulatory mechanism for c-Jun in malignant melanoma, which is summarized as a graphical abstract in Figure 4. C-Jun, monomeric TUB1A and IPO13 bind to each another in the cytoplasm of melanoma cells. Formation of this complex stabilizes c-Jun protein and is required for its nuclear translocation and activity. The complex assembles in a nuclear localization sequence (NLS)-dependent manner. Based on our latest findings and the homology of the regulatory regions shared by AP-1 family members [27], other AP-1 transcription factors could follow the same identified mechanism for nuclear translocation. Moreover, this molecular regulatory mechanism could also be present in other cancer cell types. Thus, targeting specific molecules stabilized by microtubules controlling cell proliferation and differentiation could lead to the development of improved chemotherapeutics against cancer [28].

## 3. Materials and Methods

### 3.1. Cells and Cell culture 

Melanoma cells were maintained in RPMI- Media (Sigma Aldrich, Steinheim, Germany) supplemented with penicillin (400 units/mL), streptomycin (50 mg/mL), L-glutamine (300 mg/mL), 10% FCS (Fetal Calf Serum, Sigma- Aldrich, Steinheim, Germany) and split 1:5 every three days. The human melanoma cell lines Mel Juso, Mel Ju, and Mel Im were cultured as described [29]. The human cell clone Hmb2-5, which resembles human melanocytes, was generated in our laboratory [19]. A panel of Mel Ju cells was established by stable transfection with either the pGL2 reporter plasmid or the AP-1 luciferase reporter plasmid and co-transfected with pCDNA3 (Invitrogen NV Leek, Holland), containing the selectable marker for neomycin resistance. Control cells received the empty pCDNA3 plasmid. The cells were transfected using the Lipofectamine LTX (Invitrogen Groningen, The Netherlands) method. One day after transfection, the cells were placed in selection medium containing 50 mg/mL Geneticin (G418) Sigma Aldrich Deisenhofen, Germany). After 14 days of selection, individual G418-resistant colonies were subcloned. The amount of either the pGL2 reporter plasmid or the AP-1 luciferase reporter plasmid was determined by measuring the luciferase activity of the cells by a luminometric assay (dual-luciferase reporter assay; Promega, Mannheim, Germany). These cell lines were treated with G418 (2 mg/mL) once a week to ensure selection. Nocodazole and paclitaxel (Calbiochem Merck Biosciences, Darmstadt, Germany) were used as specific cytoskeleton-disrupting agents. 

### 3.2. Analysis of Gene Expression by Quantitative PCR

cDNAs of total RNA fractions were generated using SuperScript II Reverse Transcriptase Kit (Invitrogen, Groningen, The Netherlands). qRT–PCR (quantitative real time PCR) was performed on a Lightcycler (Roche, Mannheim, Germany). cDNA template (500 ng), 0.5 μL (20 μM) of forward and reverse primers and 10 μL of SybrGreen LightCycler Mix in a total of 20 μL were applied to the following PCR program: 30 s at 95 °C (initial denaturation); 20 °C/s temperature transition rate up to 95 °C for 15 s, 3 s at 62 °C, 5 s at 72 °C, 81 °C acquisition mode single, repeated 40 times (amplification). The PCR reaction was evaluated by melting curve analysis and determining the PCR products on agarose gels, applying specific sets of primers. β-Actin or GAPDH were used for normalization. Primers sequences are listed in the Table 1 below. 

### 3.3. Western Blot Analysis

To obtain whole cell protein lysates, 3 × 106 cells were resuspended in 200 μL RIPA buffer (Roche) and lysed for 15 min at 4 °C. Insoluble fragments were removed by centrifugation at 13,000 r.p.m. for 10 min at 4 °C and the supernatant was stored at 20 °C. Western blot analyses were performed as described previously [25]. Briefly, 20–40 μg of RIPA complete cell lysates was loaded per lane and separated on SDS–PAGE (sodium dodecyl sulfate polyacrylamide gel electrophoresis) gels (Invitrogen, Carlsbad, CA, USA) and subsequently blotted onto a PVDF (polyvinylidene difluoride) membrane. After blocking for 1 h with 5% BSA/TBS-T (Phosphate buffered saline/Tris-buffered saline plus Tween 20) in case of anti-c-Jun, anti-Karyopherin 13 (IPO13), anti-LAMIN, anti-H2A, and anti-TUB1 and 5% non-fat dry milk/Tris-buffered-saline-Tween-20 (TBS-T) in the case of anti-CYLD and anti-HA-tag, the membrane was incubated for 16 h with one of the following antibodies: anti-c-Jun (1 in 1000 dilution; Cell Signaling, Frankfurt am Main, Germany), anti-β-ACTIN (1 in 5000 dilution; Sigma-Aldrich, Steinheim, Germany), anti-TUB1 (1 in 2000 dilution; Millipore, Billerica, MA, USA), anti-HA-tag (1 in 1000 dilution; Cell Signaling), anti-LAMIN (1 in 500 dilution; Millipore), anti-H2A (1 in 1000 dilution; Cell Signaling), and anti-Karyopherin 13 (IPO13) (1 in 1000 dilution; Santa Cruz, CA, USA). After three washing steps with TBS-T (0.1%), the membrane was incubated for 1 h with an alkaline phosphate coupled secondary anti-mouse (1 in 3000 dilution in TBS-T), anti-rabbit (1 in 3000 dilution in TBS-T), anti-rat (1 in 3000 dilution in TBS-T), or anti-goat IgG (Immunglobulin G) antibody (Chemicon, Hofheim, Germany), and then washed again three times in TBS-T. Finally, immunoreactions were visualized by NBT/BCIP (Sigma-Aldrich) staining. 

### 3.4. Co-Immunoprecipitation

Mel Juso or Mel Ju cells (3 × 10^6^) were lysed in 100 μL of RIPA buffer (Roche) and incubated for 15 min at 4 °C. Insoluble fragments were removed by centrifugation at 13,000 r.p.m. for 10 min at 4 °C, and the supernatant was stored at −20 °C. Protein-G-sepharose beads (GE Healthcare, Munich, Germany) were rinsed four times with ice-cold PBS (used in all subsequent washing steps) and incubated with 100 μg of pre-cleared protein at a total volume of 500 μL at 4 °C overnight. The protein-linked G-sepharose beads were incubated with anti-c-Jun, anti-TUB1, anti-CYLD, anti-Karyopherin 13 (IPO13), or anti-β-ACTIN primary antibodies at 4 °C overnight. The beads were washed four times with ice-cold PBS and resuspended in 30 μL of 4 × Roti^®^Load (Carl Roth GmbH + Co. KG, Karlsruhe, Germany). Each experiment was repeated at least three times. 

### 3.5. Reporter Gene Analysis

A total of 800,000 cells (in case of 96h transfection experiments) or 2 × 10^5^ cells (in the case of 24 h transfection experiments) were seeded into each well of a six-well plate and transfected with 0.5 μg of reporter constructs using Lipofectamine LTX (Invitrogen). For co-transfection experiments, 0.5 μg of expression plasmid or related empty vector were transfected in addition to the reporter constructs. The cells were lysed with Passive Lysis Buffer 1× (Promega, Mannheim, Germany) 24–96 h after transfection, and luciferase activity was determined. To normalize transfection efficiency, 0.2 μg of a pRL-TK plasmid (Promega, Mannheim, Germany) was co-transfected in each sample reaction and Renilla luciferase activity was measured by a luminometric assay (dual-luciferase reporter assay; Promega, Mannheim, Germany). Each experiment was repeated at least three times. 

### 3.6. Gene Suppression Using siRNA

siRNA transfection of Mel Juso cells was performed using the reverse transfection protocol of the Lipofectamine RNAiMAX reagent (Invitrogen, Carlsbad, CA, USA) according to the manufacturer’s instructions. Eight times, 104 cells were transfected with 10 nM of Tub1A (Hs_TUBA3_2; Qiagen, Germantown, MD, USA) and 40 nM of IMPORTIN13 siRNAs (Biomers.net GmbH, Ulm, Germany) or negative control siRNA (Qiagen, Germantown, MD, USA) for 96 h. Each experiment was repeated at least three times. 

### 3.7. Spin-Down Assay

The microtubule spin-down assay was performed using a Microtubule Binding Protein Spin-down Assay Kit (BK029; Cytoskeleton, Denver, CO, USA) according to the manufacturer’s instructions. Mel Im protein lysates were pre-centrifuged to prevent the protein from pelleting in the absence of polymerized microtubules. The supernatant and pellet were combined with 4× Roti^®^Load buffer (Carl Roth GmbH + Co. KG, Karlsruhe, Germany), and equal amounts of sample were loaded on a 12.5% SDS-PAGE gel for analysis by western blotting. 

### 3.8. Immunofluorescence Staining

Cells (5 × 10^4^ each well) were seeded on four-well culture slides (Becton Dickinson Labware, Franklin Lakes, NJ, USA). After 24 h, slides were washed in PBS, fixed with 4% paraformaldehyde for 30 min and non-specific binding was blocked using 1% BSA/PBS. Slides were then incubated with an anti-c-Jun antibody (1:40; Santa Cruz Biotechnology, Heidelberg, Germany) or anti-TUB1 antibody (1:500; Sigma-Aldrich, Steinheim, Germany), washed three times with PBS, and incubated with the AlexaFlour-anti-rabbit or AlexaFlour-anti-mouse antibody (1:150; Invitrogen, Groningen, The Netherlands). Afterwards, they were washed again and finally sealed with VectaShield mounting medium (Vector Laboratories, Burlingame, CA, USA) including 1 mg/mL DAPI (Sigma-Aldrich, St. Louis, MO, USA). Images were collected by fluorescence microscopy or confocal microscopy.

### 3.9. Gel Shift Experiments

Nuclear extracts of Mel Juso and Mel Ju cells were prepared and a double stranded oligomeric binding site for AP-1 (5′-CGC TTG ATG AGT CAG CCG GAA-3′; Promega, Mannheim, Germany) was phospholabeled and used for gel mobility shift assays, as described previously [20,30]. For gel shifts, an anti-c-Jun antibody (Upstate, Merck, Darmstadt, Germany) and an anti-TUB1 antibody (Millipore, Billerica, MA, USA) were used. 

### 3.10. Mutation of the c-Jun Nuclear Localization Sequence

The human c-Jun expression vector (Ha-tagged c-Jun Mut miR-125b; [25]) was used for the mutagenesis study. This vector led to the expression of stable c-Jun because the miR-125b seed sequence was destroyed. To destroy the c-Jun nuclear localization sequence (c-Jun NLS), 10 nucleotides were exchanged using a QuickChange site-directed mutagenesis kit (Stratagene, La Jolla, CA, USA) according to the manufacturer’s instructions. The primers used, MUTPrimerNLS, are listed in Table 1. 

### 3.11. Paclitaxel Treatment of Melanoma Bearing Mice

Seven -week-old Melanoma-bearing *ret* transgenic mice [24] were treated with 15 mg/kg of paclitaxel (two injections of paclitaxel at day 0 and 5). Control mice received solvent (PBS) only. Mice were sacrificed at day 7. Mouse experiments were carried out within the framework of the animal application with the number 35-9185.81/G-67/13 (Regierungspräsidium Karlsruhe, Germany).

### 3.12. Melamoma Tissues from Patients before and after Paclitaxel Treatment

Paraffin-embedded metastatic melanoma tissue of five patients before and after treatment with paclitaxel (Taxol^R^) was obtained from the SCABIO tissue bank at the Department of Dermatology, West German Cancer Center, University Hospital Essen. The sampling and handling of patient material was carried out in accordance with the ethical principles of the Declaration of Helsinki and IRB approval (protocol code: #12-5152-BO).

### 3.13. Immunohistochemical Analysis

Human metastatic melanoma tissue and murine melanoma tissues were screened for c-Jun protein expression by immunohistochemistry. The samples were prepared as described previously [31]. The tissues were incubated with primary monoclonal rabbit anti-c-Jun antibody (1:400; Cell Signaling, Frankfurt am Main, Germany). Immunohistochemical analyses were performed by an experienced pathologist (R.K.). 

### 3.14. Statistical Analysis

Results are expressed as means ± s.d. (range). Comparison between groups was made using the Student’s unpaired *t*-test or two-way ANOVA as appropriate. A *p*-value of <0.05 was considered statistically significant (*: *p <* 0.05; ns: not significant). Densitometric analysis was performed using LabImage 1D (Kapelan Bio-Imaging, Leipzig, Germany). Whole cell protein expression was normalized to β-Actin or GAPDH and nuclear protein expression to H2A or LAMIN. All calculations were performed using the GraphPad Prism Software (GraphPad Software, Inc., San Diego, CA, USA).

## 4. Conclusions

The results of our study provide novel insights into molecular functions of microtubules and their interacting partners in malignant melanoma. These findings enlarge our understanding of the mechanism of action of microtubule-targeting drugs and may help to improve their therapeutic success. Furthermore, they indicate novel therapeutic targets and prognostic markers for malignant melanoma and potentially also other types of cancer.

## Figures and Tables

**Table 1:** Primers for mRNA quantification by qRT-PCR and mutation studies.

Gene	Primer Nucleotide Sequence (fwd/rev)
β-actin	5′-CTACGTCGCCCTGGCTTCGAGC-3′ 5′-GATGGAGCCGCCGATCCACACGG-3′
importin β/ karyopherin	5’-CAGCAGAACAAGGACGGCCCC-3′ 5′-TGCTGCTTTGCAGGGGTTCCA-3′
importin 7	5′-AGTGAGTGGCGCTATTCCTG-3′ 5′-CCCTGGTGCTGTTTCTCGAT-3′
importin 8	5′-GAACCTCCACCAGGAGAAGC-3′ 5′-AGCTTGCACTGCTCTGTGAT-3′
importin 9	5′-AATTCAGACCAGGCTCACCG-3′ 5′-AGGCGGGGCAAAATAATCCA-3′
importin 13	5′-TTCCCTGAGGCACCTACTGT-3′ 5′-GCCTCCTTGATCCACATGCT-3′
α-tubulin	5′-GAAGCAGCAACCATGCGTGA-3′ 5′-GTGCCAGTGCGAACTTCATC-3′
MUTPrimer NLS c-Jun R273A/R275A	5′-CCTCCAAGTGCGCGAAAGCGAAGCTGGAGAGAATCGCCC-3′ 5′-GGGCGATTCTCTCCAGCGCCGCCGCCGCGCACTTGGAGG-3′
MUTPrimer NLS c-Jun K274A/K276A	5′-GCCTCCAAGTGCGCGGCGGCGGCGCTGGAGAG-3′ 5′-CTCTCCAGCGCCGCCGCCGCGCACTTGGAGGC-3′

## Supplementary Materials

The following are available online at https://www.mdpi.com/2072-6694/11/11/1806/s1, Figure S1: Microtubule targeting drugs (MTDs) influence AP-1 activity and AP-1 DNA-binding activity, Figure S2: Co-sedimentation by ultra-centrifugal spin-down assays show no binding between c-Jun and polymerized microtubules, Figure S3: Determination of the input protein amounts used for immunoprecipitation (experiments shown in Figure 2a,b and Figure 3d–h), Figure S4: Determination of TUB1A-suppression efficiency by qRT-PCR and Western blot analysis, Figure S5: α-Tubulin knockdown resulted in a reduced AP-1 activity in a stable AP-1 LUC clone, Figure S6: c-Jun, but not TUB1A, is involved in the AP-1-DNA binding complex, Figure S7: Determination of Importin si-RNA transfection efficiency by qRT-PCR and Western blot analysis, Figure S8: Control immunoprecipitations (IP).
