# Supplementary material for: Complex Formation with Monomeric α-Tubulin and Importin 13 Fosters c-Jun Protein Stability and Is Required for c-Jun’s Nuclear Translocation and Activity
Source: Cancers (Basel). 2019 Nov 17;11(11):1806. doi: 10.3390/cancers11111806 (PMC6895814; doi:10.3390/cancers11111806)
Supplement: Supplementary file 1 [file cancers-11-01806-s001.pdf]

Supplementary files

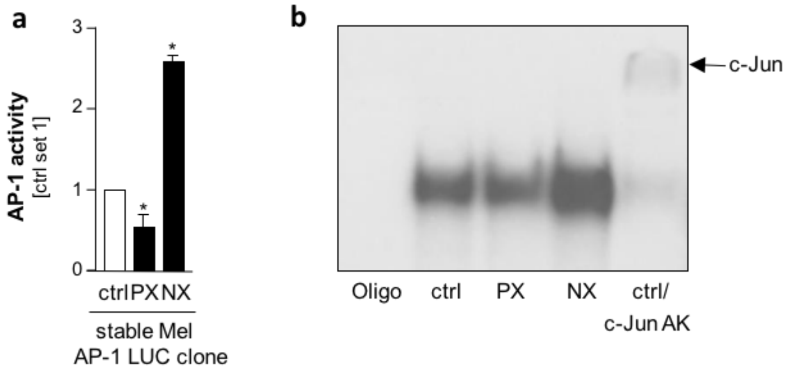

Supplementary Figure S1: Microtubule targeting drugs (MTDs) influence AP-1 activity and AP-1 DNA-binding activity. (a) AP-1 luciferase reporter gene assays with a stable Mel Ju AP-1 LUC cell clone and the control clone (stable Mel Ju pGL2) after incubation with paclitaxel (PX; 10 μM) or nocodazole (NX; 30 μM). Bars show the means ± s.d. of three independent experiments (*:$P<0.05$ compared to ctrl). (b) EMSA with nuclear extracts of melanoma cells (Mel Juso) treated with DMSO, PX (5 μM) or NX (30 μM) using the classical AP-1 consensus sequence (AP-1 Oligo). Supershift experiments with an anti-c-Jun antibody show the direct involvement of c-JUN in the AP-1-DNA-binding complex. The experiment was repeated three times.

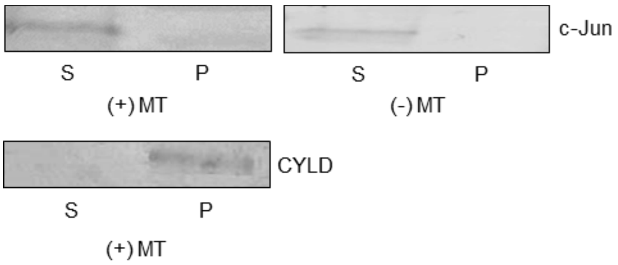

Supplementary Figure S2: Co-sedimentation by ultra-centrifugal spin-down assays show no binding between c-Jun and polymerized microtubules. Western blot analysis of the supernatant (S) and cell pellet (P) of Mel Im protein lysates after microtubule spin-down assay. In the microtubule-positive samples (+MT), c-Jun was not detectable in the pellet, and direct interactions between c-Jun and polymerized microtubules can therefore be excluded. The microtubule-negative samples (-MT) served as a negative control. CYLD served as a positive control for microtubule interaction[26]. The experiment was repeated three times.

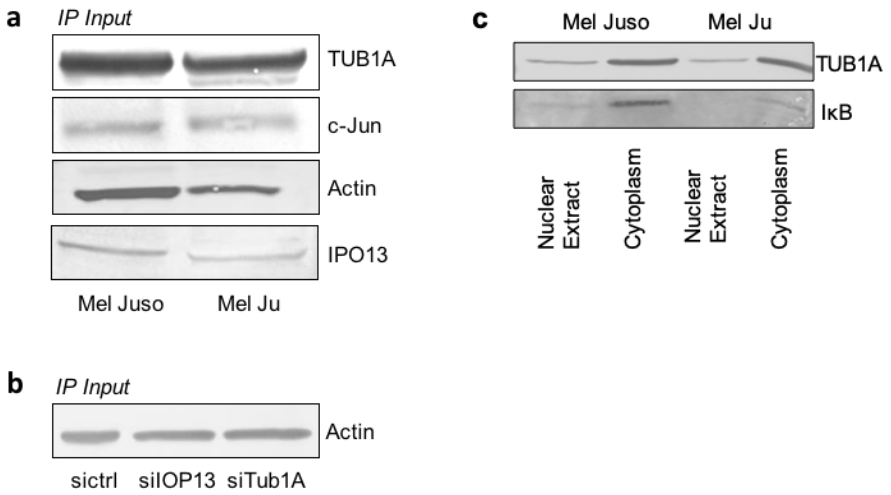

Supplementary Figure S3: Determination of the input protein amounts used for immunoprecipitation (experiments shown in Fig. 2a, 2b, 3d-h). Western blot analyses show the Input protein amounts for immunoprecipitations (IP) of **(a)** the melanoma cell lines Mel Juso and Mel Ju and **(b)** of Mel Juso Input protein amounts after sictrl, siIPO13 or siTub1A transfection. **(c)** Western blot analyses of TUB1A in nuclear extracts and cytoplasmic fractions of Mel Juso and Mel Ju cells, respectively. IkappaB was used as a loading control.

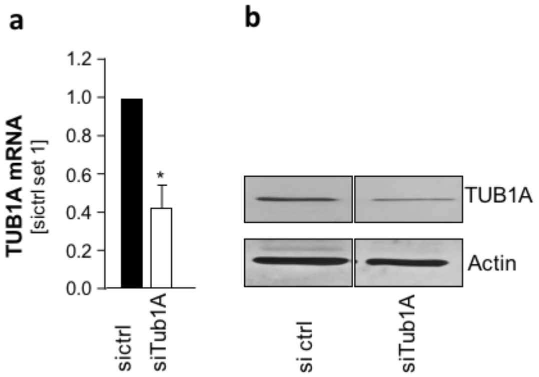

Supplementary Figure S4: Determination of TUB1A-suppression efficiency by qRT-PCR and Western blot analysis. (a) Quantification of the mRNA expression of TUB1A in Mel Juso cells after 96 h of TUB1A si- RNA (siTub1A) and control si-RNA (sictrl) transfection by qRT-PCR. Decreased levels of TUB1A mRNA after siTub1A transfection could be detected compared to the control transfected cells. Bars show the means ± s.d. of three independent experiments; (*: $P<0.05$ compared to sictrl). **(b)** Western blot analysis showing protein expression of TUB1A in Mel Juso cell lysates after siTub1A transfection. Reduced amounts of TUB1A protein were observed after siTub1A transfection compared to control-transfected cells. β-Actin was used as a loading control. The measurements were performed in replicates and each experiment was repeated at least three times.

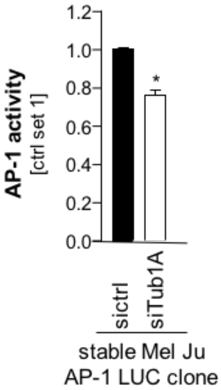

Supplementary Figure S5: α-Tubulin knockdown resulted in a reduced AP-1 activity in a stable AP-1 LUC clone. AP-1 luciferase reporter gene assays after transfection with α-Tubulin siRNA (siTub1A) compared to control transfected cells (sictrl) of stable transfected Mel Ju cells (Mel Ju (pGL2/AP-1) LUC cell clones) with AP-1 LUC constructs. Bar graph shows the mean± s.d. of three independent experiments; (*: P<0.05 compared to sictrl).

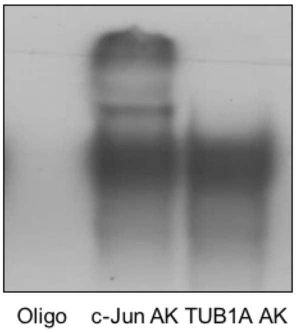

Supplementary Figure S6: c-Jun, but not TUB1A, is involved in the AP-1-DNA binding complex. EMSA with nuclear extracts of Mel Juso cells using the classical AP-1 consensus sequence (Oligo). Supershift experiments with an anti-c-Jun antibody and an anti-TUB1A antibody show the direct involvement of c-Jun in the AP-1-DNA-binding complex, but no involvement of TUB1A. The experiment was repeated three times.

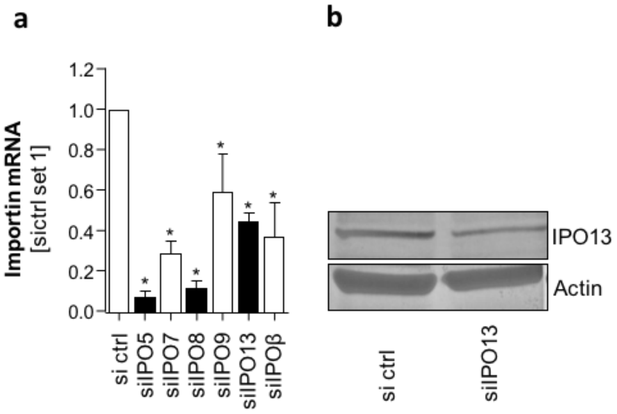

Supplementary Figure S7: Determination of Importin si-RNA transfection efficiency by qRT-PCR and Western blot analysis. (**a**) Quantification of the expression of Importin mRNA (IPO7, IPO8, IPO9, IPO13, IPOβ) in Mel Juso cells after 96 h of Importin si-RNA and control si-RNA (sictrl) transfection by qRT-PCR. The levels of all Importins decreased in the siRNA-transfected cells compared to control-transfected cells. Bars show the means ± s.d. of three independent experiments; (*: P<0.05 compared to sictrl). (**b**) Western blot analysis showing the expression of IPO13 in Mel Juso cell lysates after IPO13

si-RNA transfection. Reduced amounts of IPO13 protein were observed after IPO13 si-RNA transfection compared to control transfected cells. β-Actin was used as a loading control. The measurements were performed in replicates and each experiment was repeated at least three times.

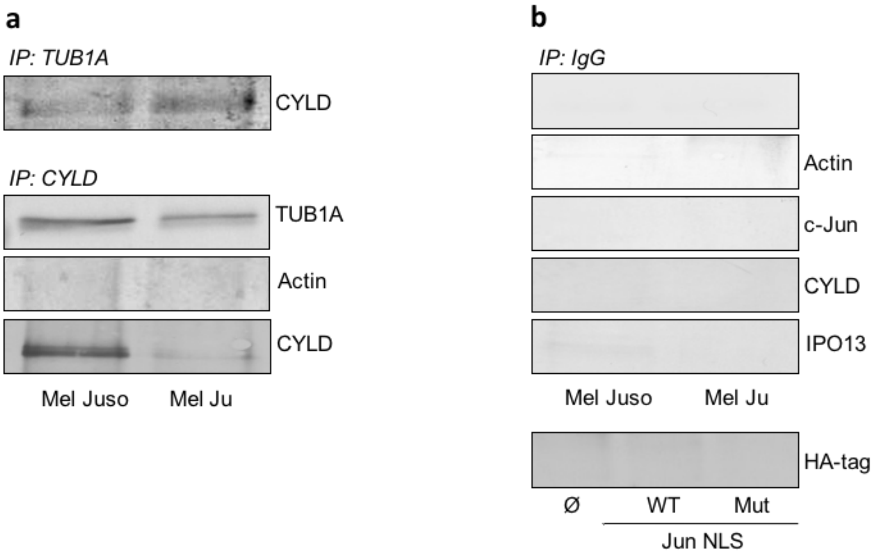

Supplementary Figure S8: Control immunoprecipitations (IP). (**a**) Western Blot analysis after co-immunoprecipitation with an anti-CYLD or anti-TUB1A antibody, respectively, showed an interaction between CYLD protein and TUB1A[26]. (**b**) Co-immunoprecipitation with an anti-IgG antibody served as a negative control for all performed co-immunoprecipitations. Western Blot analysis after co-immunoprecipitation with an anti-IgG antibody showed no detectable protein amount of TUB1A, Actin, c-Jun, CYLD, IPO13 or HA-tagged c-Jun, respectively. Each experiment was repeated at least three times.

Full unedited gel for Figure 1b:

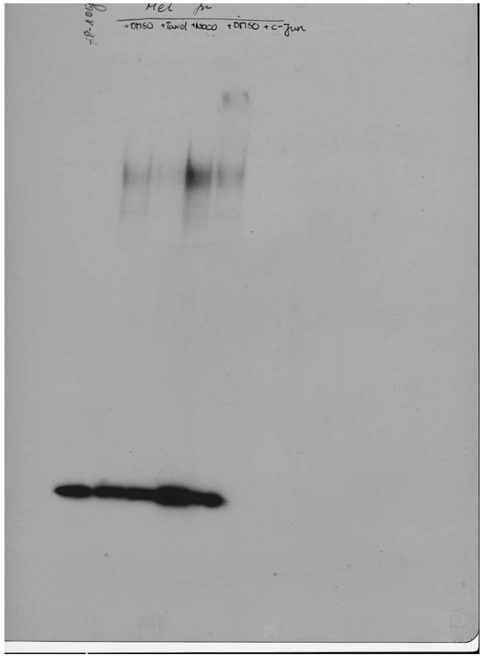

SS

AP-1 Oligo

DMSO

paclitaxel

nocodazole

DMSO /c-JUN AK

Mel Ju

Full unedited gel for Figure S1b:

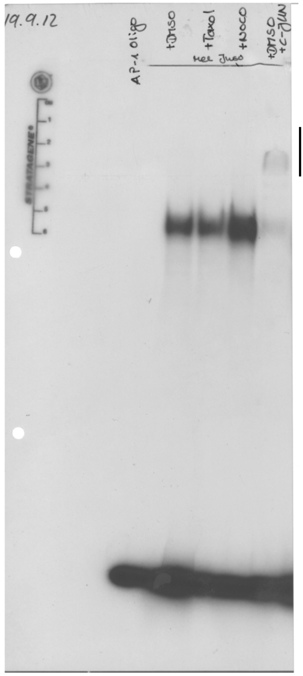

SS

AP-1 Oligo

DMSO

paclitaxel

nocodazole

DMSO /c-JUN AK

Mel Juso

Full unedited gel for Figure 1c:

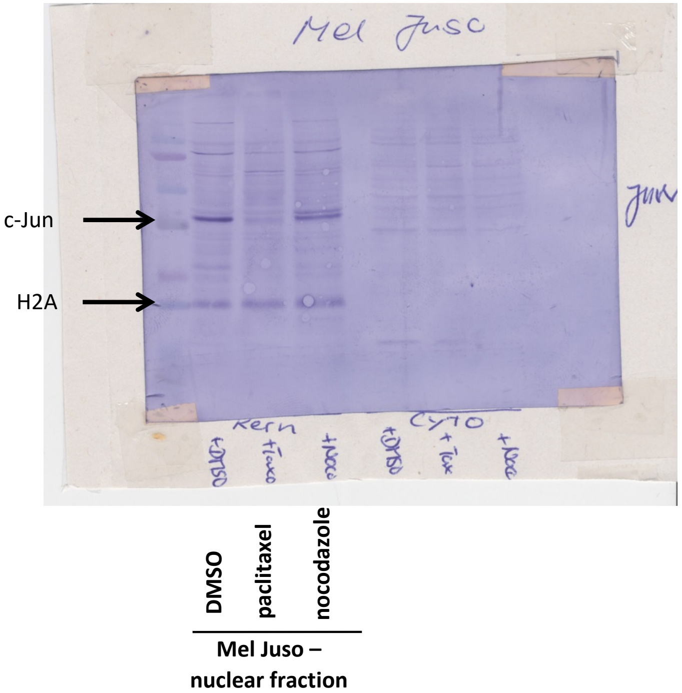

Protein Standard:

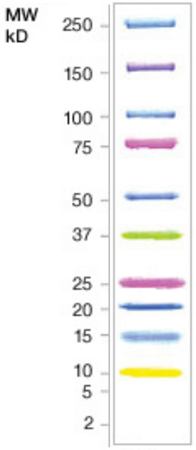

Full unedited gel for Figure S3:

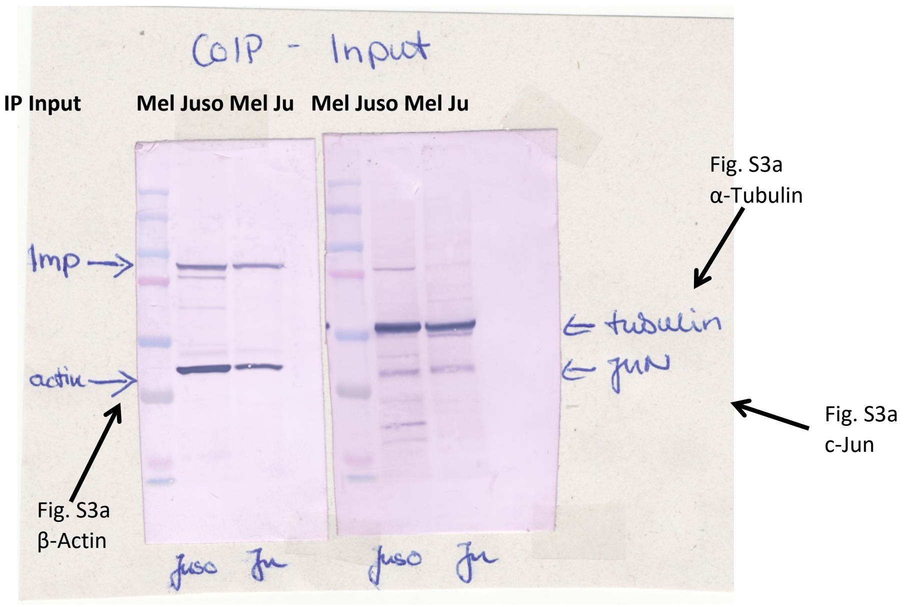

Full unedited gel for Figure 3B:

Note: antibody heavy chain and light chain bands at ~25, 50 kDa

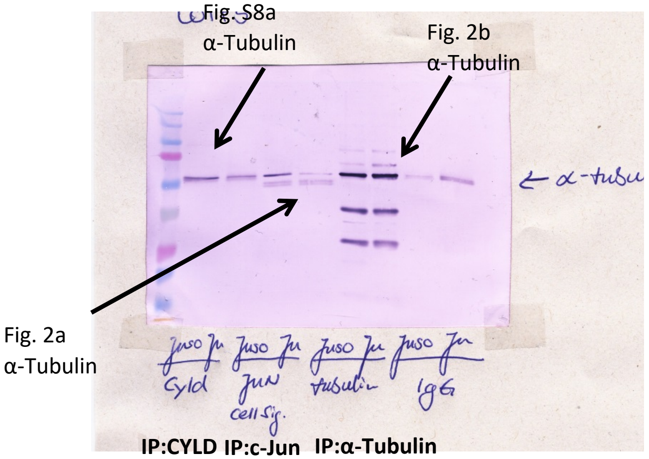

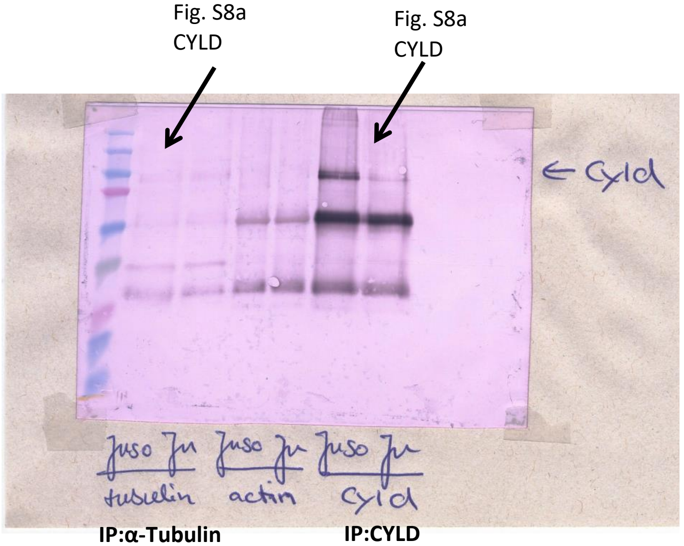

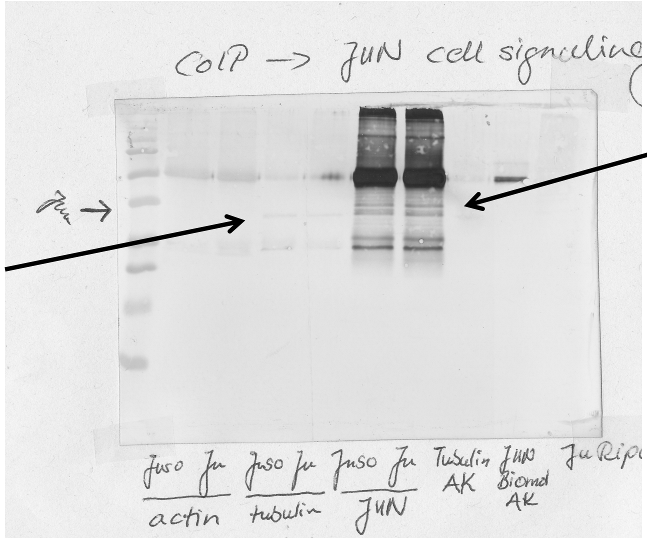

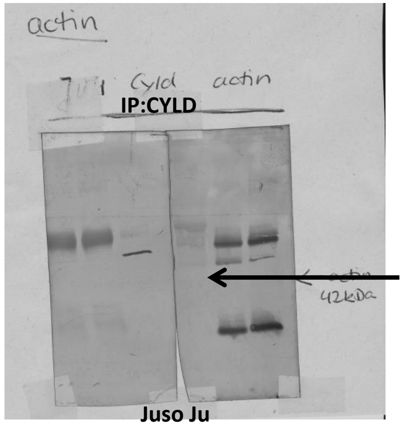

Full unedited gel for Figure 2d:

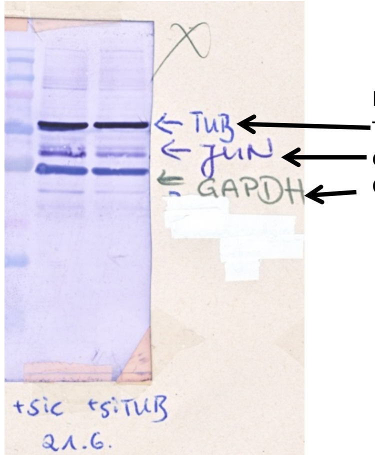

Fig. 2d:
Tubulin
c-Jun
GAPDH

sictrl siTub1A

Mel Juso
protein lysate

Full unedited gel for Figure 2e:

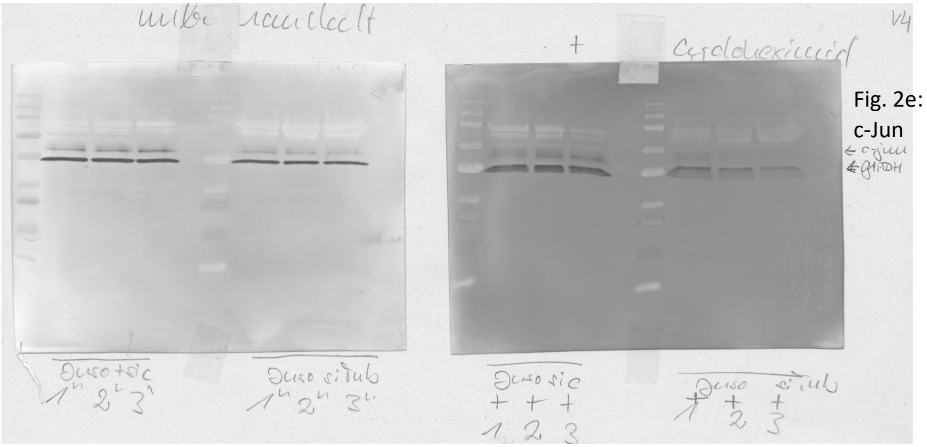

Full unedited gel for Figure 2f:

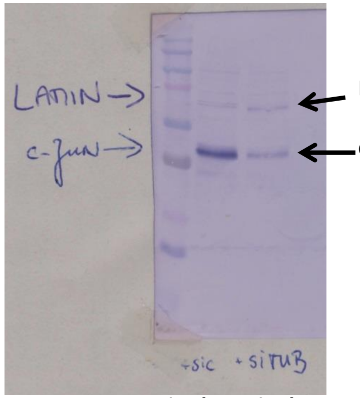

Lamin
c-Jun

sictrl siTub1A

Mel Juso
nuclear extract

Full unedited gel for Figure 3b; 3c:

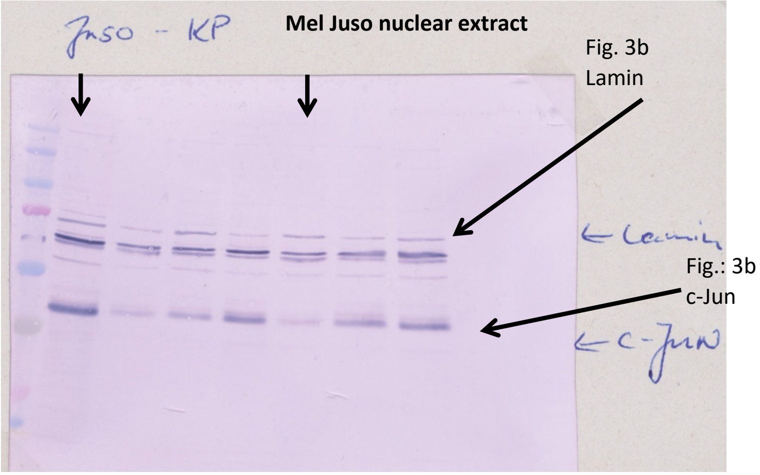

+sictrl.

+siIPO13

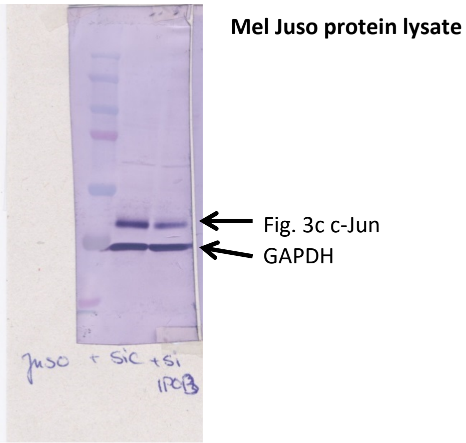

Full unedited gel for Figure 3 d, e, f:

Fig. S 3a
IPO13

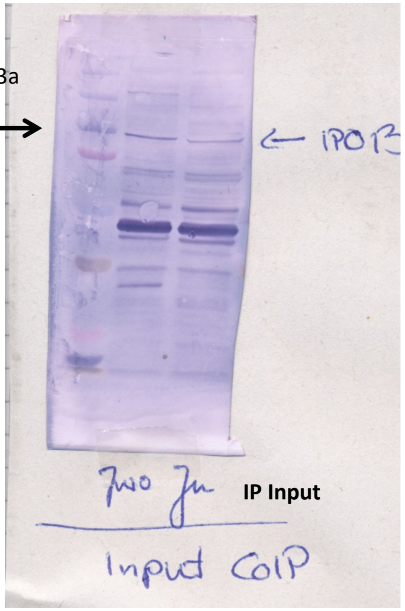

Fig. 3e
IPO13

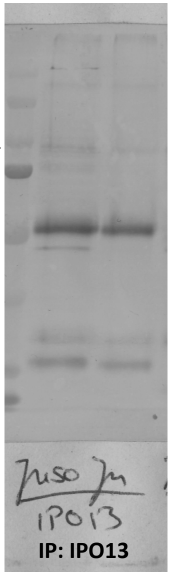

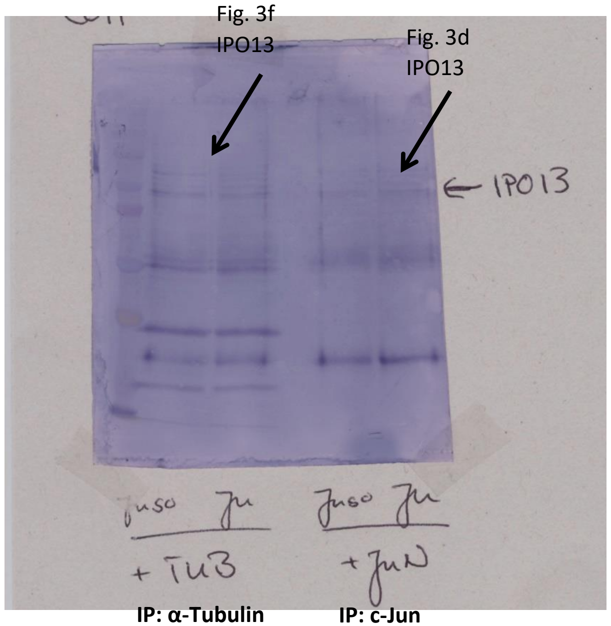

IP: α-Tubulin　　IP: c-Jun

Fig. 3e
α-Tubulin

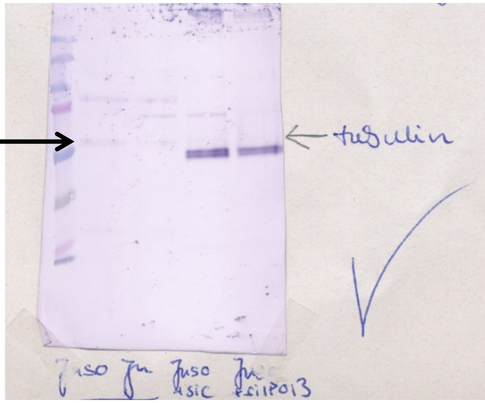

IP: IPO13

Full unedited gel for Figure 3 d, f:

Fig. 3d
c-Jun

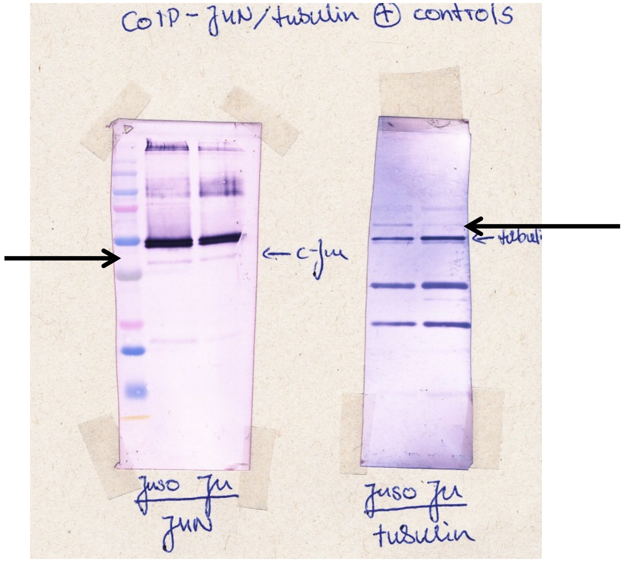

Fig. 3f
α-Tubulin

IP: c-Jun **IP: α- Tubulin**

Full unedited gel for Figure 3g:

IP: c-Jun

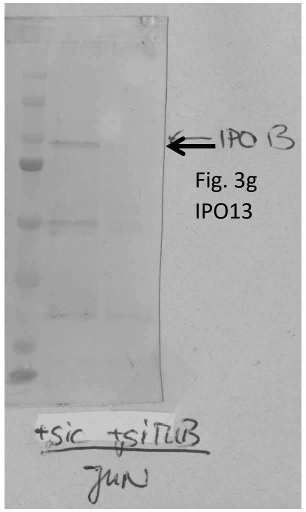

sictrl siTub1A

Mel Juso

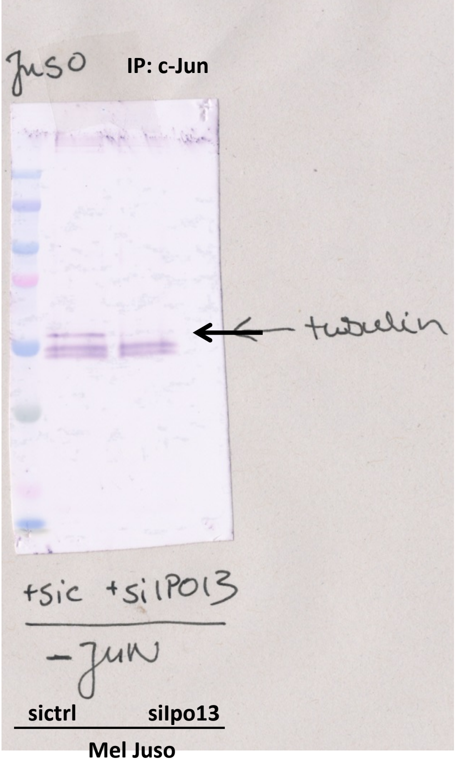

Mel Juso

Fig. 3g
α-Tubulin

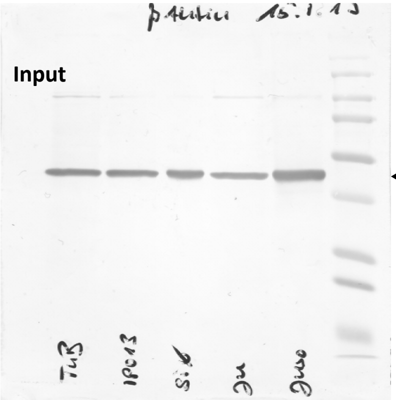

Fig. S3b
β-Actin

siTub1A siIpo13 sictrl.

Mel Juso

Full unedited gel for Figure 3h:

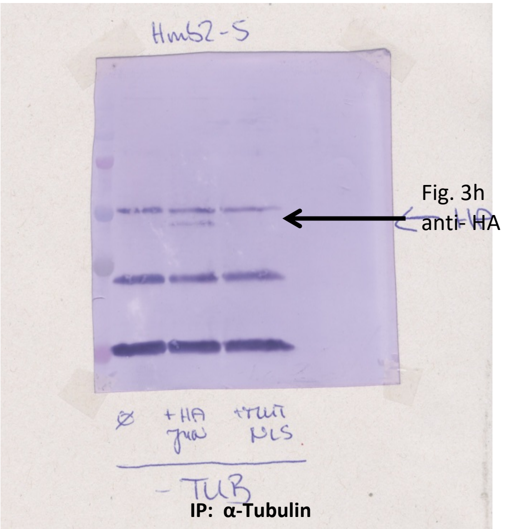

IP: α-Tubulin

Jun WT NLS

Jun MUT NLS

Hmb2-5

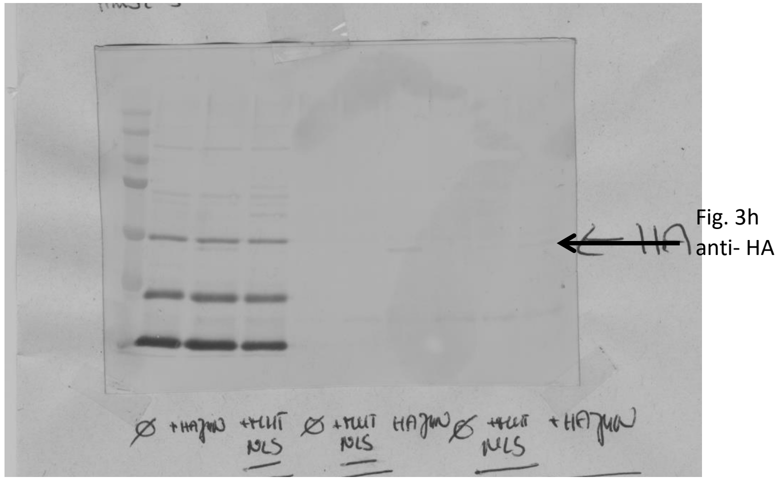

IP: IPO13

Jun MUT NLS

Jun WT NLS

Hmb2-5

Full unedited gel for Figure 3 i, j:

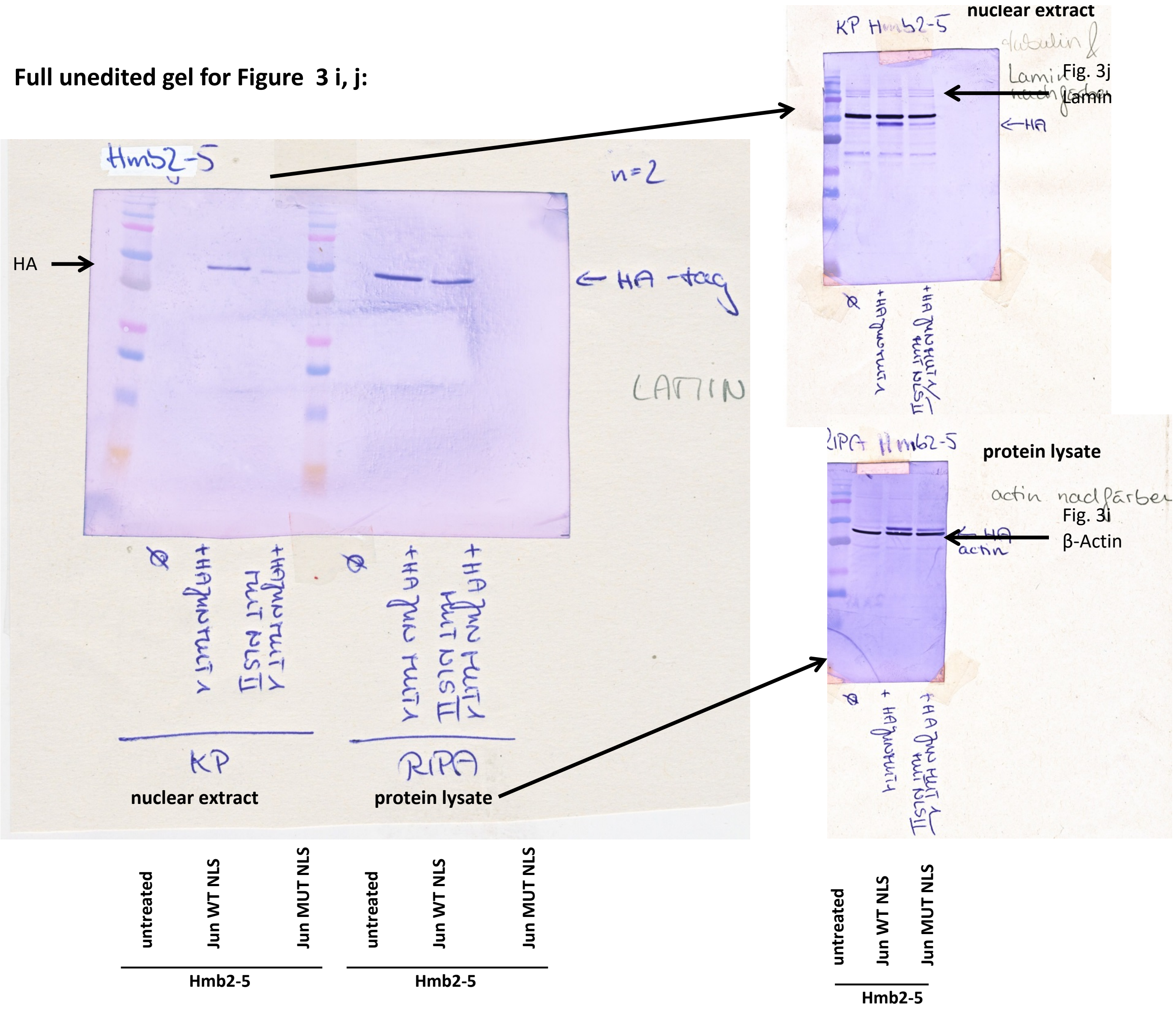

Full unedited gel for Supplement Figure S2:

c-Jun

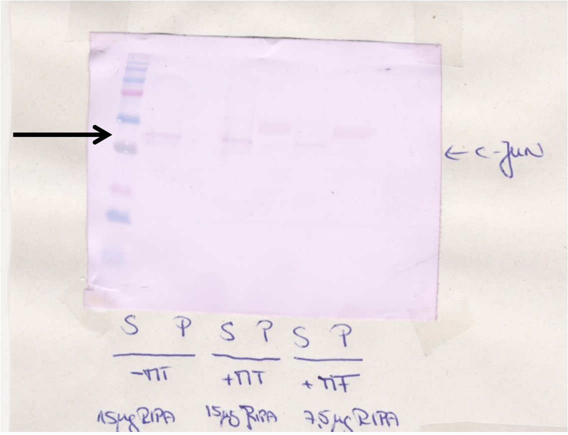

S P S P
(-)microtubule (+)microtubule

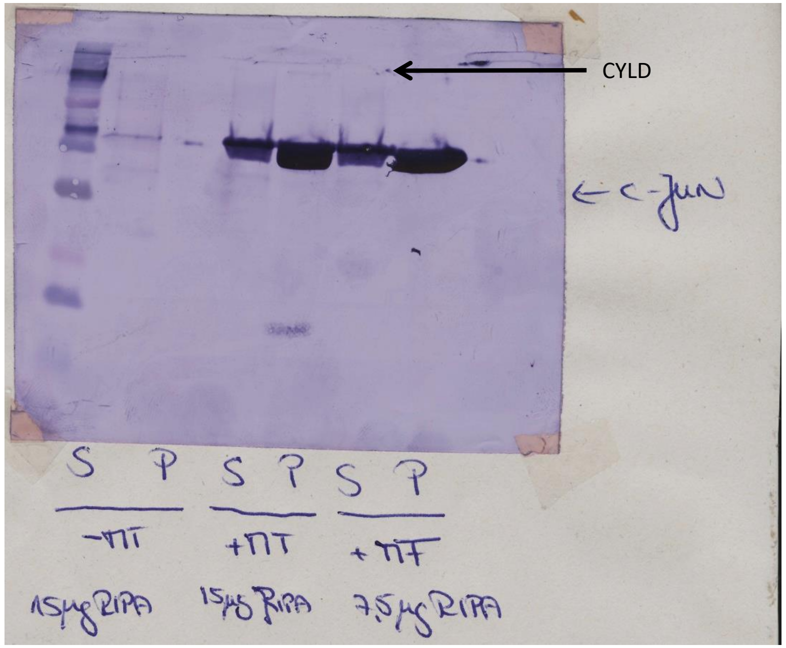

S P
(+)microtubule

Full unedited gel for Figure Supplement Figure S4 a,b:

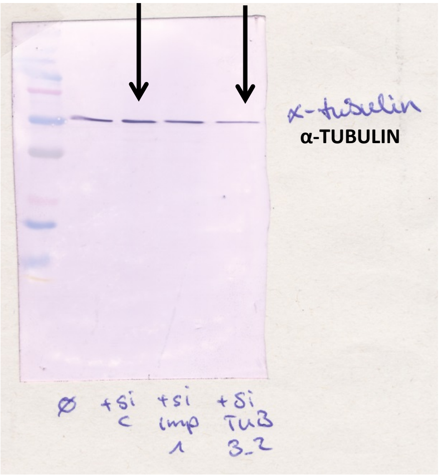

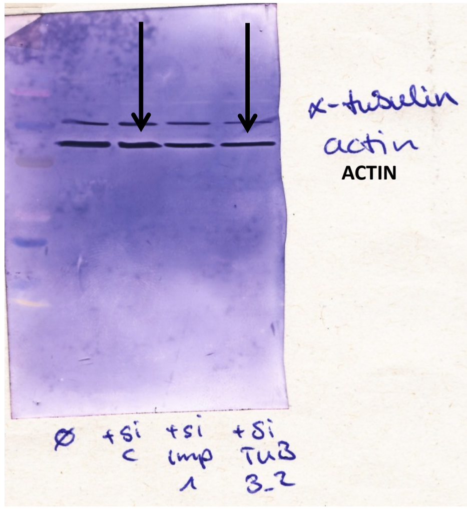

si ctrl

siTub1A

Mel Juso

Full unedited gel for Figure Supplement Figure S7:

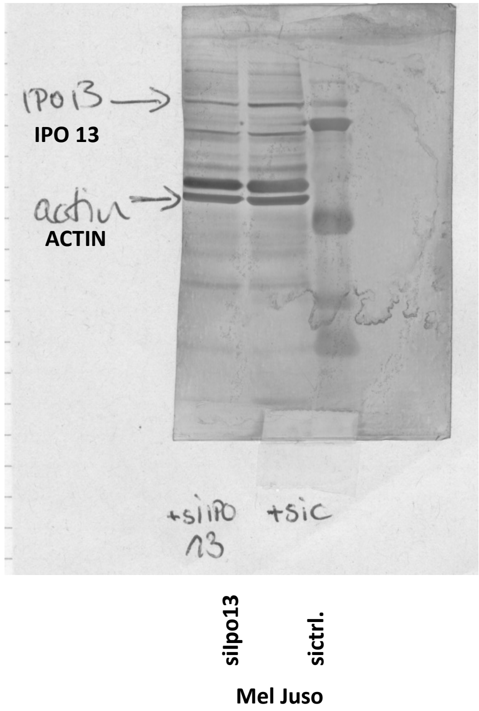

Full unedited gel for Figure Supplement Figure S8a,b:

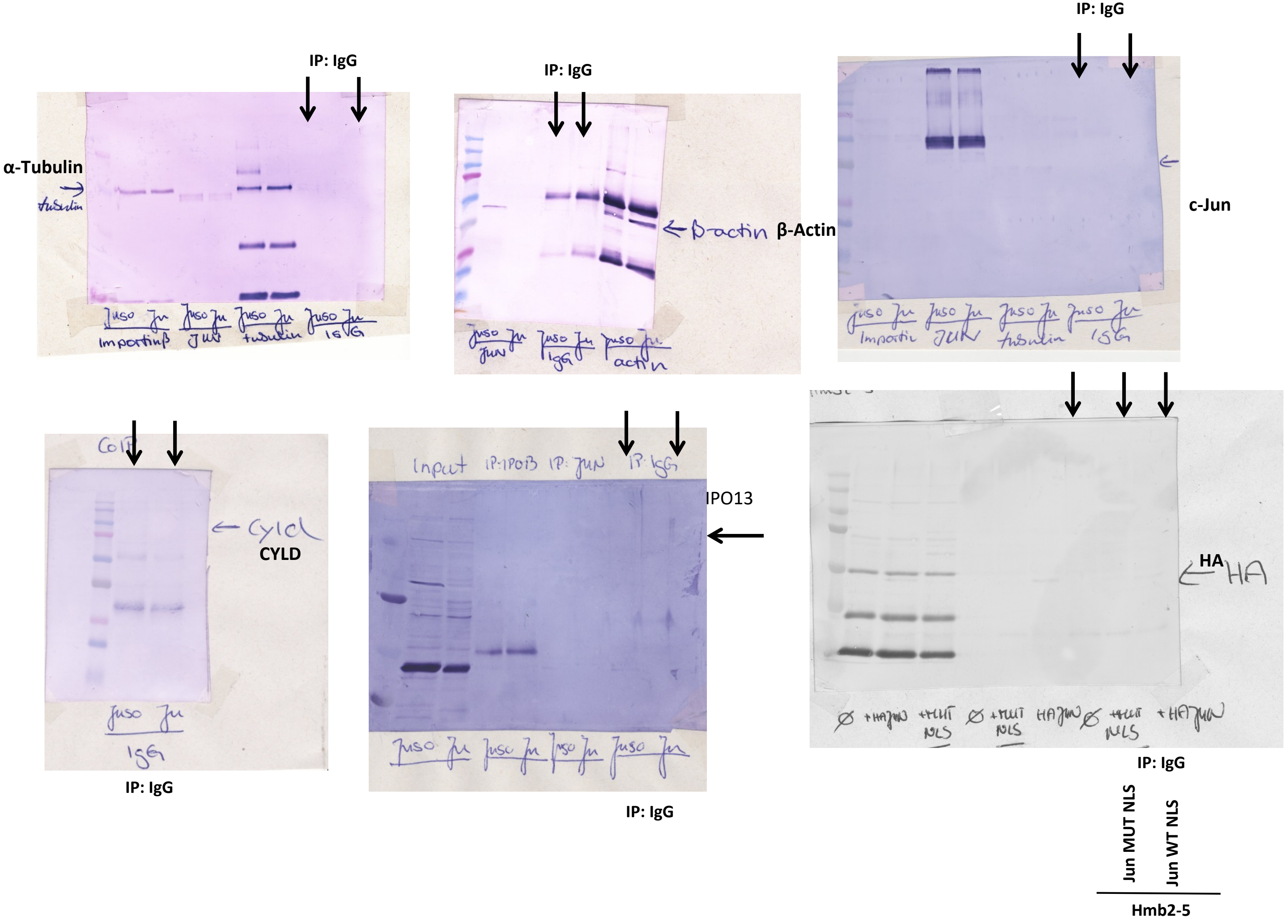

Full unedited gel for Figure Supplement Figure S8 a,b:

c-Jun

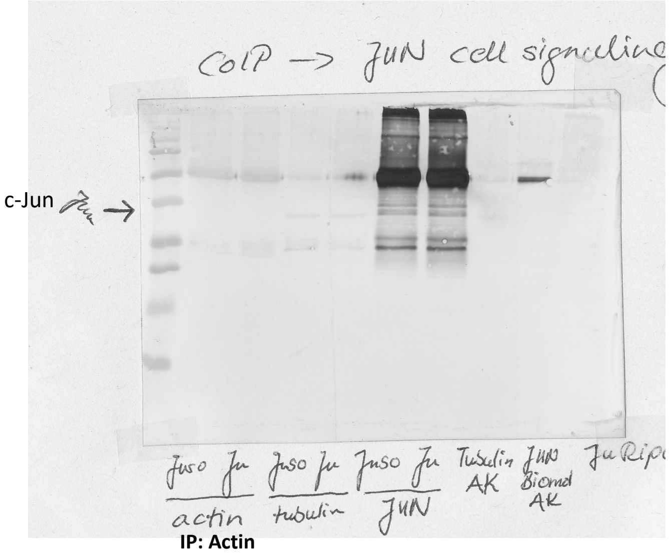

IP: Actin

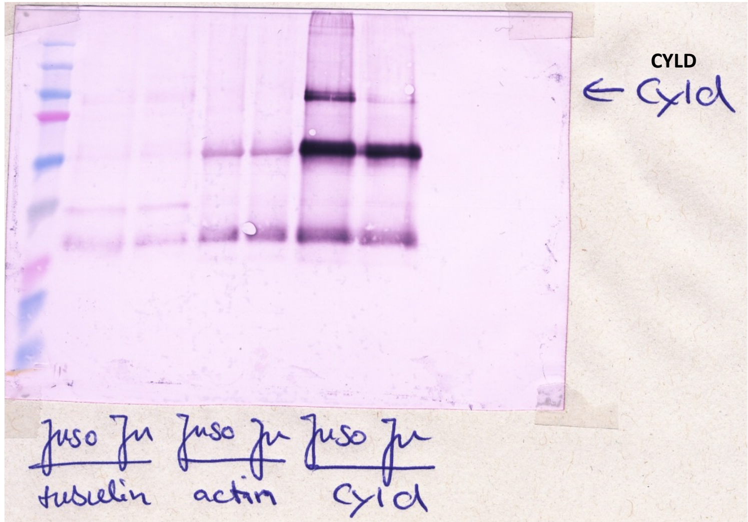

CYLD

IP: Actin

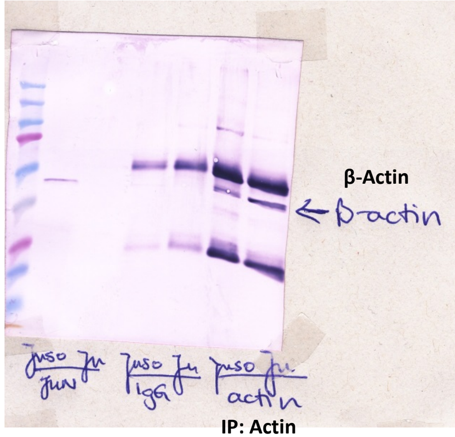

β-Actin

IP: Actin
